# Machine learning detection of obstructive hypertrophic cardiomyopathy using a wearable biosensor

**DOI:** 10.1038/s41746-019-0186-x

**Published:** 2019-12-10

**Authors:** R. Ueno, H. Matsui, L. Xu

**Affiliations:** 1 Frankston Hospital, Melbourne, Australia; 2 The University of Tokyo, Tokyo, Japan

**Keywords:** Diseases, Computational biology and bioinformatics, Diagnosis

**Arising From** Green et al. *npj Digital Medicine* 10.1038/s41746-019-0130-0 (2019)

Green et al. (June 24 issue)^1^ developed a machine learning classifier of hypertrophic cardiomyopathy (HCM) patients using a noninvasive optical sensor incorporated in commercial smart watches. The study included 83 patients (19 patients with HCM and 64 healthy controls) and a machine learning classifier was trained with Leave-One-Group-Out cross-validation with nested hyperparameter tuning, achieving a C-statistic of 0.99 (95% CI: 0.99–1.00) for 82 patients.

While these results are promising and persuasive enough to facilitate further trial to assess its performance in a larger cohort, we would argue a few statistical concerns with the study. First, the authors argued that they have performed 68-fold, instead of 82-fold cross-validation for their Leave-One-Group-Out cross-validation. Further clarification might be required to avoid any confusion for readers. Second, the confusion matrix includes all the 82 patients from both train and test data, while this table should ideally include only test data.^2^ Inclusion of train group in this matrix might result in the over-estimate performance of the model.

We believe those findings might not invalidate the insightful work by Green et al. Further clarification of such concerns might further reinforce this study to function as a foundation for a larger study.
